# Challenges in managing a multifactorial eosinophilic pneumonia: daptomycin vs strongyloidiasis case report

**DOI:** 10.1186/s12879-022-07852-y

**Published:** 2022-11-22

**Authors:** Lynda G. J. Eckhardt, Jordan L. Kelley, Dorothy Maes

**Affiliations:** 1 Department of Pharmacy Services, University of Kentucky HealthCare Good Samaritan Hospital, 310 South Limestone, B003, Lexington, KY 40536 USA; 2 University of Kentucky College of Pharmacy, 789 South Limestone, Lexington, KY 40536 USA; 3 Department of Pulmonary, Critical Care and Sleep Medicine, University of Kentucky Medical Center, Kentucky Clinic, Room K504, 740 South Limestone Street, Lexington, KY 40536 USA

**Keywords:** Eosinophilia, Daptomycin, Strongyloides, Corticosteroids, Pulmonary

## Abstract

**Background:**

Eosinophilia is defined as a blood eosinophil count > 500/mcL with etiology usually an allergic reaction or parasitic infection which can lead to serious organ damage.

**Case presentation:**

A patient being treated for hardware infection develops eosinophilia while on daptomycin in the setting of a positive strongyloides antibody. The patient was on chronic steroids prior to admission for epitheliopathy which complicated care. The daptomycin was discontinued, ivermectin initiated to treat strongyloidiasis, and high dose steroids initiated simultaneously. Eosinophilia resolved and patient discharged home after two months in the hospital.

**Conclusion:**

Multifactorial eosinophilia poses question of steroid harm in the setting of parasitic infection. Patient was treated for both strongyloides and daptomycin induced eosinophilia with improvement and discharge from the hospital.

## Background

Eosinophilia should be investigated when moderate elevation is present, especially with various etiologies [1]. Eosinophilia, defined as a blood eosinophil count > 500/mcL and significant organ damage, can occur with moderately elevated eosinophil counts > 1500/mcL due to the inflammatory reaction [2]. Daptomycin is a cyclic lipopeptide antimicrobial that provides coverage against gram positive bacteria [3]. Daptomycin has the potential for developing acute eosinophilic pneumonia from macrophages acting as eosinophilic chemoattractants [4]. Strongyloides is a nematode and a common cause of eosinophilia in immunocompromised patients [5]. Below we present a case report of a patient with multifactorial eosinophilia that was treated for strongyloidiasis and given steroids for daptomycin induced eosinophilia. Opportunistic infections and hypersensitivity reactions have conflicting treatments and could lead to clinical inertia. The patient achieved complete recovery within 48 h of treatment with resolution of eosinophils.

## Case presentation

**Fig. 1:**
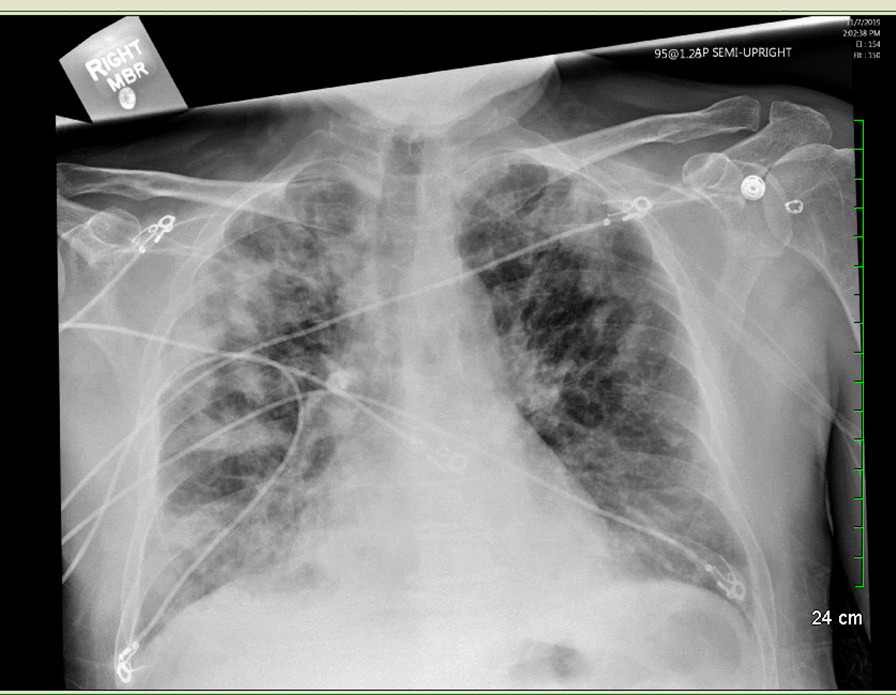
Chest X-ray

**Fig. 2:**
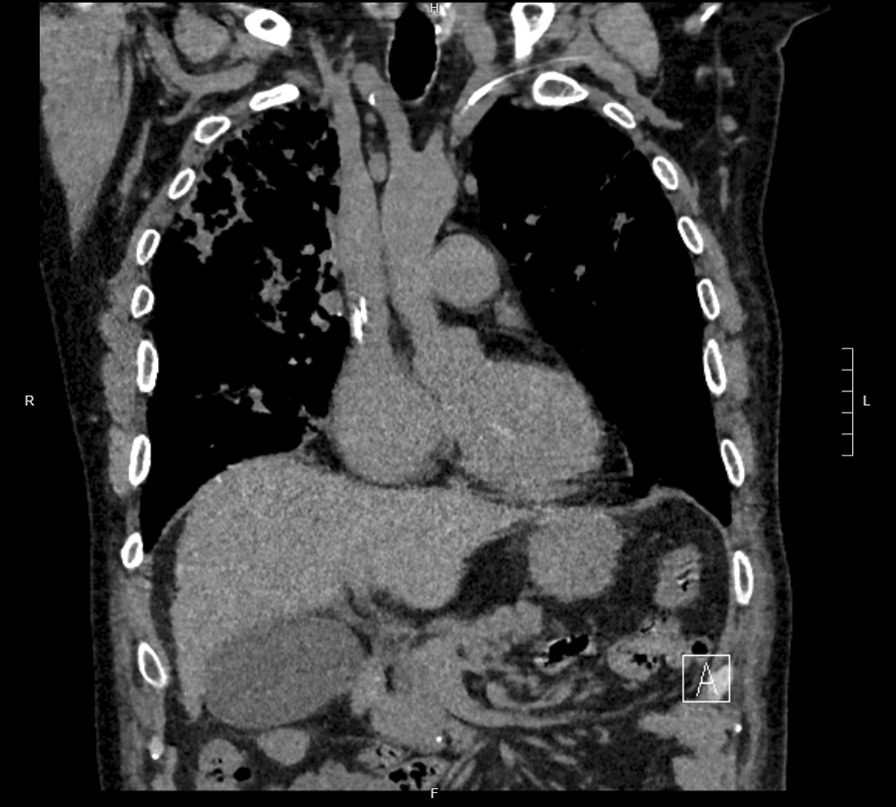
CT high resolution—chest

**Fig. 3:**
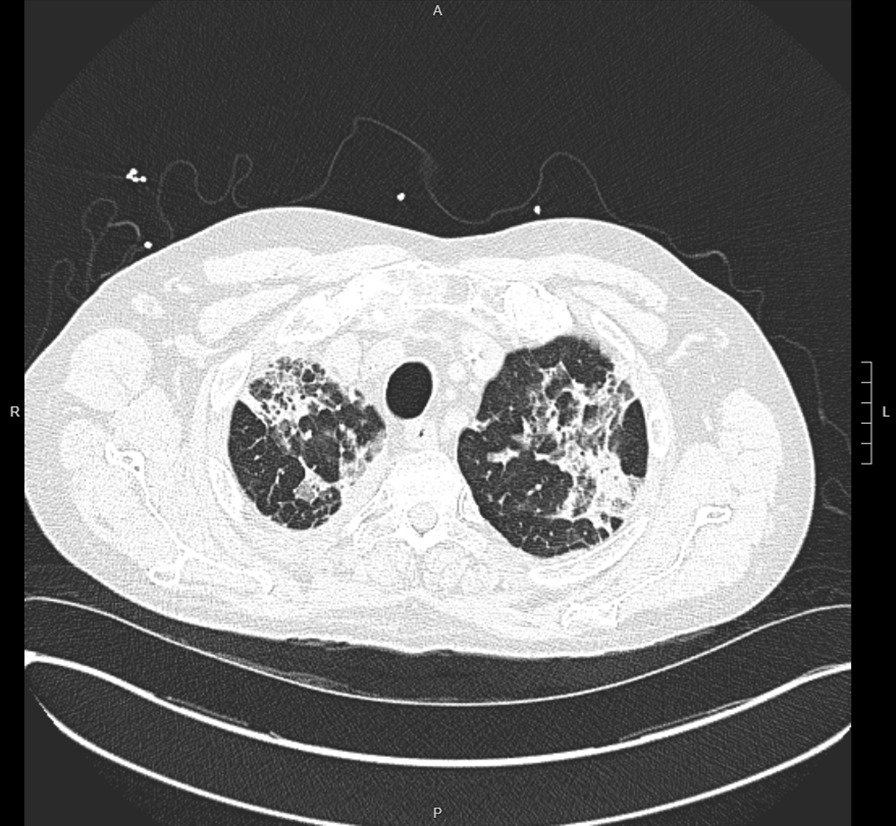
CT high resolution—chest

A patient with cirrhosis, atrial fibrillation, and acute posterior multifocal placoid pigment epitheliopathy (APMPPE) presents with drainage from right lower extremity (RLE) splint following open reduction and internal fixation (ORIF) for an ankle fracture. Given the recent surgery, elevated inflammatory markers, and one week history of foul smelling drainage from surgical incisions, there was concern for right ankle surgical site infection (SSI) with probable implant infection given retained hardware. Patient was initiated on vancomycin, cefepime and metronidazole for broad spectrum coverage pending culture results. Orthopedic surgery was consulted to assess the need for surgical intervention. While standard of care is to remove the implant, removal would have been suboptimal from a surgical perspective. The decision was made to leave the device in place and treat with antimicrobials for six weeks. Intraoperative cultures were positive for pan-sensitive *Enterococcus faecalis, Alcaligenes faecalis* (variable resistance), *Staphylococcus epidermidis*, and *Corynebacterium jeikeium*. Antimicrobial therapy was switched to daptomycin for better coverage of *E. faecalis*, and meropenem for coverage of the *A. faecalis*. The patient was on chronic glucocorticoid treatment (prednisone 60 mg daily) for APMPPE; therefore, after discussions with ophthalmology, it was decided to taper the steroids off due to improvement in vision in the setting of an acute SSI. Prednisone taper was initiated on hospital day 5 (decreasing by 10 mg every 4 days until off), with the last dose being administered on hospital day 26. On day 28 of admission, patient developed eosinophilia with a rise in absolute eosinophilic count (AEC) from 0.3 k/µL (4%) on day 1 to an AEC of 2.42 k/µL (17%) on day 28. The patient began to clinically decompensate with worsening dyspnea and new oxygen requirements resulting in their transfer to the intensive care unit (ICU) for management of acute decompensated respiratory failure. The differential diagnosis included strongyloidiasis and daptomycin-induced eosinophilic pneumonia. Chest radiography showed patchy bilateral airspace disease, greatest on the right, and pulmonary vascular congestion (Fig. 1). Computerized tomography (CT) scan of chest showed apical predominant bilateral patchy ground glass opacities with associated consolidation and septal thickening (Figs. 2 and 3). Out of concern for daptomycin-induced pneumonitis, therapy was changed back to vancomycin, which the patient continued for the remainder of the treatment course. The patient received a total of 29 doses of daptomycin, with the last dose administered on day 35 of admission. No further daptomycin was administered to the patient. The patient underwent bronchoscopy on day 37, which was grossly normal. Bronchoalveolar lavage (BAL) showed 10% eosinophils and 30% lymphocytes. A transbroncial lung biopsy (TBLB) was also performed, with pathology revealing eosinophils present but not increased, and no organisms were seen in the tissue. Total immunoglobulin E (IgE) was 633 kU/L (< 214 kU/L), Aspergillus IgE was negative, and strongyloides antibody was significantly elevated at 1.5 Index Value (< 0.9 IV). Ivermectin was initiated for treatment of strongyloides at 200 µg/kg × 2 doses. The patient was started on prednisone (1 mg/kg/day) for 3 days after completing ivermectin, with plans to continue prednisone 40 mg daily. Steroids were discontinued when the patient improved clinically and esosinophilia resolved (Table 1) given concern for impaired healing of RLE fracture. No further imaging was performed due to futility in the setting of clinical improvement. Patient completed antimicrobial therapy inpatient without further complications and was subsequently discharged home.

**Table 1:** Eosinophilia trend

Hospital day	Eosinophils
1	0.3 k/µL (4%)
21	0.18 k/µL (1%)
28	2.42 k/µL (17%)
32	2.34 k/µL (17%)
35	2.48 k/µL (15%)
37	1.81 k/µL (12%)
39	0.97 k/µL (10%)
41	0.84 k/µL (9%)
49	0.34 k/µL (4%)

## Discussion and conclusions

Eosinophils are multifunctional leukocytes produced from the multipotent hematopoietic stem cells that synthesize cytokines and growth factors responsible for homeostatic processes and inflammatory regulation [6]. Eosinophilia is usually defined by a blood eosinophil count > 500/µL commonly occurring in the presence of an allergic reaction. More severe presentations include drug reaction with eosinophilia and systemic symptoms (DRESS) or parasitic infection. Significant organ damage can occur with moderately elevated eosinophil counts > 1500/µL due to inflammatory reaction. Most commonly impacted organs are heart and lungs [2]. Eosinophilia should be investigated regardless of signs and symptoms of associated organ involvement when moderate elevation is present [1, 6]. Daptomycin induced eosinophilic pneumonia and strongyloides are commonly included in the differential workup for patients with eosinophilia. [7]

Daptomycin is a lipopeptide antimicrobial that gained drug approval in the United States (U.S.) in 2003. The activity of daptomycin is dependent on free calcium to insert and disrupt the integrity of the gram positive plasma membranes leading to cell death [3, 8]. Onset of eosinophilic pneumonia associated with daptomycin use is exposure dependent, documented to occur at 2.8 ± 1.6 weeks [7]. Eosinophilic pneumonia is rare but is thought to be caused from macrophages leading to an inflammatory response with eotaxin, an eosinophil chemoattractant. There is some speculation that since daptomycin binds to human surfactant, it accumulates in the alveolar space leading to injury and inflammation [7]. In 2010, the U.S. Food and Drug Administration (FDA) released a safety warning for daptomycin induced eosinophilic pneumonia leading to symptoms of fever, dyspnea, and new infiltrates on chest X-ray (Table 2) [4]. Case reports and series recommend a short course of steroids to decrease the inflammatory pathway and discontinuation of the offending agent [9]. As patient had been on chronic steroid therapy prior to admission, the eosinophil response could have been blunted [10].

**Table 2:** Diagnostic criteria for daptomycin or drug induced eosinophilia

FDA guidance for attributing eosinophilic pneumonia to daptomycin	Solomon and Schwartz criteria for drug or toxin induced eosinophilic pneumonia
Exposure to daptomycin	Presence of eosinophilic pneumonia on lung biopsy or BAL (> 25%) in the setting of parenchymal infiltrates
Fever	Presence of a potential candidate drug or toxin in an appropriate time frame
Dyspnea with increased oxygen requirement (or mechanical ventilation requirement)	No other cause of eosinophilic pneumonia such as fungal or parasitic infection
New infiltrates on chest X-ray or computed tomography (CT) scan	Clinical improvement after cessation of the drug or toxin
Bronchoalveolar lavage (BAL) with > 25% eosinophils	Recurrence of eosinophilic pneumonia with rechallenge to the drug or toxin
Clinical improvement following daptomycin withdrawal	

Strongyloides (*Strongyloides stercoralis*) induced eosinophilia is thought to be caused by the immunomodulatory process induced by parasites. In fact, most parasites present with some form of eosinophilia. Nematodes are microscopic plant feeding roundworms with a six stage life cycle [11]. Around 50% of the human population worldwide have gastrointestinal (GI) nematode infections attributed to poor sanitation practices. Strongyloidiasis is caused by an intestinal nematode acquired by walking barefoot in the soil. Hyperinfection can occur in immunocompromised patients or those on chronic steroids [12]. Strongyloides can be diagnosed via blood test or the larvae can be seen in the stool when examined under a microscope [13]. Strongyloides is usually a chronic parasitic infection due to a phenomenon known as autoinfection, where a complete parasitic life cycle occurs within a single organism without the involvement of another host, which can be treated with ivermectin or albendazole [5].

Eosinophilia can be correlated with significant allergic reactions or parasitic infections. Above we describe a patient with eosinophilia of unknown origin in the setting of daptomycin and strongyloides. The patient was treated with ivermectin for strongyloides and steroids for the daptomycin induced eosinophilia. The patient began to improve immediately and was subsequently discharged home. The patient was immunocompromised due to chronic steroid use which could have explained the pathogenicity of strongyloides. It is difficult to decipher the true cause of eosinophilia in this case which led to a multifactorial approach. Patient comorbidities and symptoms often obscure the differential diagnosis, compelling clinicians to prioritize treatment based on the most urgent need. In this case, removing the offending agent by discontinuing daptomycin, treating the strongyloidiasis, and adjusting steroids for the treatment of eosinophilia was the course of action selected.

An additional confounder in this case was that the patient was being treated for APMPPE with oral prednisone 60 mg daily (~ 0.6 mg/kg/day), which was initiated 35 days prior to hospital admission. Steroids are commonly used for ocular diseases due to their multimodal approach as an anti-inflammatory and angiostatic properties. Local steroids may also be used to reduce systemic effects and prevent toxicities [14]. There is not current consensus on how to treat APMPPE, however steroids, specifically glucocorticoids, are postulated to help with vasculitis and foveal involvement. Systemic steroids (1 mg/kg/day oral prednisolone) are generally initiated in the acute phase of APMPPE and continued until vision improvement. Glucocorticoids are suggested to cause retinal cell death through various pathways, which is why long term steroid use is not ideal and the steroids are typically tapered off over several weeks [15]. Eosinophil reduction with steroids is an inverse, dose-dependent relationship. Time to reduction of eosinophils can range from days to weeks depending on dose response. Steroids can be given concomitantly with antimicrobials with appropriate monitoring and low risk of septicemia [16].

In conclusion, this patient had a multifactorial eosinophilia in the setting of chronic steroids which complicates all aspects of medical care. The associated findings on CT are also non-specific and not diagnostic of either differential diagnosis. This is a complicated patient population where we have to question how to treat strongyloidiasis in an immunocompromised patient who would otherwise benefit from a higher dose of steroids. Disseminated strongyloidiasis carries a high mortality rate especially in those who are immunocompromised [13]. Ideally, the standard of care is removal of the offending agent which would grant clinicians time to treat the strongyloidiasis before initiating corticosteroids. In the patient presented above, it is more likely that strongyloidiasis was the offending agent as it is unlikely the patient had a negative strongyloides antibody on admission that converted over given the geological environment of the patient population. However, the acute increase of eosinophils in the setting of daptomycin matches the documented time of onset and exposure. Daptomycin was discontinued, the patient was treated for strongyloidiasis, and steroids were reinitiated simultaneously. The patient’s AEC decreased back to baseline and the patient was discharged home after a 2 month inpatient hospitalization.

## Data Availability

Not Applicable. Data sources included Sunrise Clinical Manager Electronic Medical Record. Authors choose not to distribute data to protect the patient’s identity in the setting of a single case report.

## Abbreviations

AEC: Absolute eosinophilic count
APMPPE: Acute posterior multifocal placoid pigment epitheliopathy
BAL: Bronchoalveolar lavage
CT: Computerized tomography
DRESS: Drug reaction with eosinophilia and systemic symptoms
FDA: Food and Drug Administration
GI: Gastrointestinal
IgE: Immunoglobulin E
IV: Index value
ICU: Intensive care unit
kg: Kilogram
mg: Milligram
ORIF: Open reduction and internal fixation
RLE: Right lower extremity
SSI: Surgical site infection
TBLB: Transbroncial lung biopsy
U.S.: United States
